# Searching for causal relationships of glioma: a phenome-wide Mendelian randomisation study

**DOI:** 10.1038/s41416-020-01083-1

**Published:** 2020-10-06

**Authors:** Charlie N. Saunders, Alex J. Cornish, Ben Kinnersley, Philip J. Law, Richard S. Houlston, Collaborators, Elizabeth B. Claus, Dora Il’yasova, Joellen Schildkraut, Jill S. Barnholtz-Sloan, Sara H. Olson, Jonine L. Bernstein, Rose K. Lai, Stephen Chanock, Preetha Rajaraman, Christoffer Johansen, Robert B. Jenkins, Beatrice S. Melin, Margaret R. Wrensch, Marc Sanson, Melissa L. Bondy

**Affiliations:** 1 Division of Genetics and Epidemiology, The Institute of Cancer Research, London, SW7 3RP UK; 2 School of Public Health, Yale University, New Haven, CT 06510 USA; 3 Department of Neurosurgery, Brigham and Women’s Hospital, Boston, MA 02115 USA; 4 Department of Epidemiology and Biostatistics, School of Public Health, Georgia State University, Atlanta, GA 30303 USA; 5 Duke Cancer Institute, Duke University Medical Center, Durham, NC 27710 USA; 6 Cancer Control and Prevention Program, Department of Community and Family Medicine, Duke University Medical Center, Durham, NC 27710 USA; 7 Department of Population and Quantitative Health Sciences and the Cleveland Center for Health Outcomes Research, Case Western Reserve University School of Medicine, Cleveland, OH 44106 USA; 8 Department of Epidemiology and Biostatistics, Memorial Sloan Kettering Cancer Center, New York, NY 10017 USA; 9 Departments of Neurology and Preventive Medicine, Keck School of Medicine, University of Southern California, Los Angeles, CA 90033 USA; 10 Division of Cancer Epidemiology and Genetics, National Cancer Institute, Bethesda, MD 20892 USA; 11 Danish Cancer Society Research Center, Survivorship, Danish Cancer Society, Copenhagen, 2100 Denmark; 12 Oncology Clinic, Finsen Centre, Rigshospitalet, University of Copenhagen, Copenhagen, 2100 Denmark; 13 Department of Laboratory Medicine and Pathology, Mayo Clinic Comprehensive Cancer Center, Mayo Clinic, Rochester, MI 55905 USA; 14 Department of Radiation Sciences, Umeå University, Umeå, 901 87 Sweden; 15 Department of Neurological Surgery, School of Medicine, University of California, San Francisco, CA 94143 USA; 16 Institute of Human Genetics, University of California, San Francisco, CA 94143 USA; 17 Sorbonne Université, Inserm, CNRS, UMR S 1127, Institut du Cerveau et de la Moelle épinière, ICM, F-75013 Paris, France; 18 AP-HP, Groupe Hospitalier Pitié-Salpêtrière, Service de Neurologie 2-Mazarin, Paris, 75013 France; 19 Section of Epidemiology and Population Sciences, Department of Medicine, Dan L. Duncan Comprehensive Cancer Center, Baylor College of Medicine, Houston, TX 77030 USA

**Keywords:** CNS cancer, Risk factors

## Abstract

**Background:**

The aetiology of glioma is poorly understood. Summary data from genome-wide association studies (GWAS) can be used in a Mendelian randomisation (MR) phenome-wide association study (PheWAS) to search for glioma risk factors.

**Methods:**

We performed an MR-PheWAS analysing 316 phenotypes, proxied by 8387 genetic variants, and summary genetic data from a GWAS of 12,488 glioma cases and 18,169 controls. Causal effects were estimated under a random-effects inverse-variance-weighted (IVW-RE) model, with robust adjusted profile score (MR-RAPS), weighted median and mode-based estimates computed to assess the robustness of findings. Odds ratios per one standard deviation increase in each phenotype were calculated for all glioma, glioblastoma (GBM) and non-GBM tumours.

**Results:**

No significant associations (*P* < 1.58 × 10^−4^) were observed between phenotypes and glioma under the IVW-RE model. Suggestive associations (1.58 × 10^−4^ < *P* < 0.05) were observed between leukocyte telomere length (LTL) with all glioma (OR_SD_ = 3.91, *P* = 9.24 × 10^−3^) and GBM (OR_SD_ = 4.86, *P* = 3.23 × 10^−2^), but the association was primarily driven by the *TERT* variant rs2736100. Serum low-density lipoprotein cholesterol and plasma HbA1C showed suggestive associations with glioma (OR_SD_ = 1.11, *P* = 1.39 × 10^−2^ and OR_SD_ = 1.28, *P* = 1.73 × 10^−2^, respectively), both associations being reliant on single genetic variants.

**Conclusions:**

Our study provides further insight into the aetiological basis of glioma for which published data have been mixed.

## Background

Although gliomas are not common, they account for ~80% of all malignant primary brain tumours.^1^ Moreover, these tumours pose a serious health burden because of the associated high case fatality and morbidity, the 5-year survival for glioblastoma (GBM), the most common histological subtype (∼45% of cases), being only 5%.^2^

Differences in the incidence of glioma between countries provide support, albeit indirectly, for lifestyle and/or environmental factors as being determinants of disease risk.^3,4^ Knowledge of specific aetiological risk factors for glioma has the potential to inform prevention strategies and reduce disease burden. While several factors have been linked to the occurrence of glioma, the only environmental factor consistently associated with risk is exposure to ionising radiation, which accounts for only a small proportion of cases.^4^ Epidemiological studies of other potential risk factors have been inconsistent, null or not independently validated.^5–12^ These observational studies are, however, prone to reverse causation, unmeasured confounding and recall bias, which can preclude causal inferences.^13^ Additionally, the high frequency of exposure ascertainment by proxy is another source of bias.^14^ Finally, the studies performed to date have had a limited scope of enquiry either examining factors that have well-established associations for other cancers or hypothesised risk factors based on limited insight into glioma biology, thereby reducing the prospects of revealing causal relationships.

**Fig. 1:**
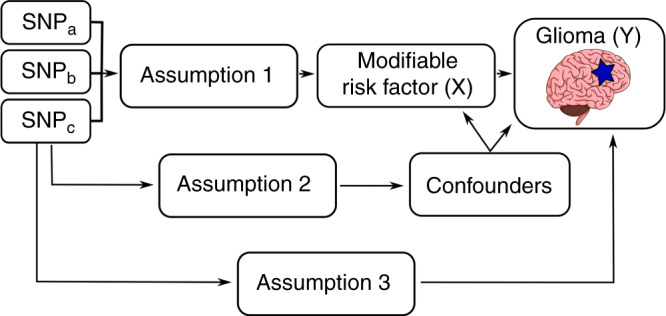
Principles of Mendelian randomisation (MR) and the assumptions required to obtain an unbiased causal effect estimate. The three assumptions are (1) genetic variants used as instrumental variables are only associated with the modifiable risk factor (X), (2) genetic variants are not associated with any measured or unmeasured confounders and (3) genetic variants only influence the risk of developing glioma (Y) through the modifiable risk factor (X). SNP single-nucleotide polymorphism.

Mendelian randomisation (MR) is an analytical approach that utilises genetic variants as instrumental variables (IVs), to assess the causal relevance of exposures in disease.^15^ Because these genetic variants are randomly assigned at conception, they are not influenced by reverse causation. In the absence of pleiotropy (i.e., variants being associated with the disease through alternative pathways), they can provide unconfounded estimates of disease risk (Fig. 1).^15^ We have previously applied MR to evaluate potential risk factors that have previously been examined in conventional epidemiological studies of glioma. Initially, we explored causal relationships with dietary factors such as vitamin D, immune response factors and obesity-related factors.^16^ After finding no strong associations, we subsequently investigated a more comprehensive list of dietary and lifestyle factors that commonly influence the risk of other cancers, again finding no evidence for strong associations.^17^ Other researchers have used MR to examine the relationship between glioma and other traits with more success, purporting a strong association with leukocyte telomere length.^18,19^ All of these MR analyses have, however, been predicated on assumptions about disease aetiology. Recently, an agnostic strategy to identify causal relationships has been proposed to examine hitherto unconsidered traits, by integrating the phenome-wide association study (PheWAS) and MR methodology, termed MR-PheWAS.^20^

To advance our understanding of the aetiological basis of glioma, we have conducted an MR-PheWAS to validate reported associations and search for a novel causal relationship that may not have been captured by previous studies. Specifically, we analysed 316 phenotypes, proxied by 8387 genetic variants, and summary genetic data from a genome-wide association study (GWAS) of glioma comprising 12,488 cases and 18,169 control subjects.^21^

## Methods

### Genetic instruments for phenotypes

Two-sample MR was conducted using the TwoSampleMR R package.^22^ Genetic instruments for the traits investigated were single-nucleotide polymorphisms (SNPs) identified from recent meta-analyses, the largest studies published to date or those curated by MR-Base (Supplementary Table A-C1). For each SNP, the chromosome position, the effect estimate expressed in standard deviations (SD) of the trait per allele and the corresponding standard error (SE) were recovered. SNPs were only considered as potential instruments if they were associated with each trait at *P* < 5 × 10^−8^ in a GWAS of European populations, and had a minor allele frequency >0.01. To avoid co-linearity between SNPs for each trait, correlated SNPs within each trait were excluded (linkage disequilibrium threshold, *r*^2^ ≥ 0.01). Only SNPs with the strongest effect on the trait were considered for the final analysis (Supplementary Table A-C2). The percentage of variance explained (PVE) by the associated SNPs was computed from the association statistics, and traits were only considered if the PVE was >0.1% and the F statistic >10 (Supplementary Table A-C1).^23,24^ We only considered continuous traits, as analysis of binary traits (such as disease status) with binary outcomes in two-sample MR frameworks is prone to inaccurate causal estimates, with bias of Wald odds ratios (ORs) of up to 76% being reported.^16,25,26^

### Glioma data

Gliomas are heterogeneous and different tumour subtypes, defined in part by malignancy grade (e.g., pilocytic astrocytoma—World Health Organization (WHO) grade I, diffuse ‘low-grade’ glioma—WHO grade II, anaplastic glioma—WHO grade III and glioblastoma (GBM)—WHO grade IV), which can be distinguished. For the sake of brevity, we considered gliomas as being either GBM or non-GBM. The association of each genetic instrument with glioma risk was examined using a summary of glioma effect estimates and the corresponding SEs from a recent meta-analysis of eight GWAS.^21^ This GWAS comprised 12,488 cases (6183 with GBM and 5820 with non-GBM pathology) and 18,169 controls of European descent (Supplementary Table A-C3).

### Mendelian randomisation analysis

The MR methodology is based on the assumption that genetic variants, used as instruments for a risk factor, are associated with the risk factor and not with confounders or alternative causal pathways (Fig. 1).^15^ Additionally, to accurately estimate the size of the causal effect, the associations must be linear and unaffected by interactions.^27^ For each SNP, causal effect estimates were generated for glioma, GBM and non-GBM tumours as ORs per one SD unit increase in the putative risk factor (OR_SD_), with 95% confidence intervals (CIs), using the Wald ratio. For traits with multiple SNPs as IVs, causal effects were estimated using a random-effects inverse-weighted variance (IVW-RE) model, which assumes that each SNP identifies a different causal effect. These causal effects are averaged to elucidate the true causal effect, due to balanced pleiotropy.^28^ To assess the robustness of our findings, we compared the causal estimates and associated *P* values using robust adjusted profile score (MR-RAPS), weighted median (WME) and weighted mode-based (WMBE) methods. For exposures with fewer than ten SNPs, the fixed-effects IVW (IVW-FE) method was utilised (Supplementary Table A-C4).^15,29,30^ We examined the potential impact of outlying and pleiotropic SNPs on causal estimates using a leave-one-out strategy, using either an IVW-RE or IVW-FE model for exposures with greater than or less than 10 SNPs, respectively (Supplementary Table A-C5). Finally, directional pleiotropy was assessed using MR-Egger regression (Supplementary Table A-C6).^31^ Heterogeneity within each trait (*I*^2^) was calculated from Cochran’s *Q* value.^32,33^ Rucker’s Q value was calculated using RadialMR (Supplementary Table A-C7).^34^

To account for multiple testing, we considered a Bonferroni-corrected *P* value of 1.58 × 10^−4^ (i.e., 0.05/316 putative risk factors) as being statistically significant. A *P* > 1.58 × 10^−4^ but <0.05 was considered to be suggestive evidence of a causal association. Statistical analyses were performed using R version 3.4.0 and MR-Base.^22,35^ Figures were generated using Inkscape version 0.92.^36^

### Estimation of study power

The power of MR to demonstrate a causal effect depends on the percentage of risk factor variance explained by the genetic variants used as instruments. We estimated the study power, stipulating an α value of 0.05 and 1.58 × 10^−4^, for each risk factor a priori across a range of effect sizes as per Brion et al. (Supplementary Table A-C1).^37^

## Results

**Fig. 2:**
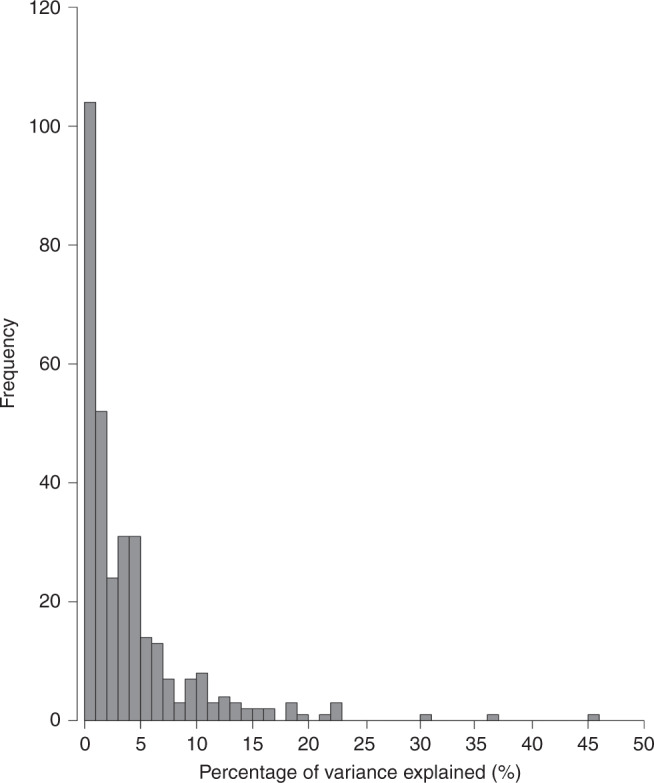
Frequency histogram of percentage of variance explained (PVE). This plot shows the PVE of single-nucleotide polymorphisms (SNP) used as instrumental variables for the 316 phenotypes.

Figure 2 shows the frequency distribution plot of the PVEs across all 316 phenotypes studied. The median PVE by SNPs used as IVs for each of the 316 phenotypes evaluated as risk factors for glioma was 2.2% (0.1–45.8%). The power of our MR study to identify causal relationships between each of the genetically defined phenotypes and glioma is detailed in Supplementary Table A1. Overall, our study had at least 80% power to detect a 1.5-fold difference in risk for 79% (251/316) of traits for all glioma. Inevitably, our power to demonstrate causal relationships for glioma subtypes was more limited. For GBM and non-GBM, we had at least 80% power to detect a 1.5-fold difference in risk for 72% (226/316) and 71% (223/316) of traits, respectively (Supplementary Table B1 and C1). The power of our study to demonstrate a causal association for glioma, GBM and non-GBM over a range of PVEs is shown in Supplementary Fig. 1.^37^

**Fig. 3:**
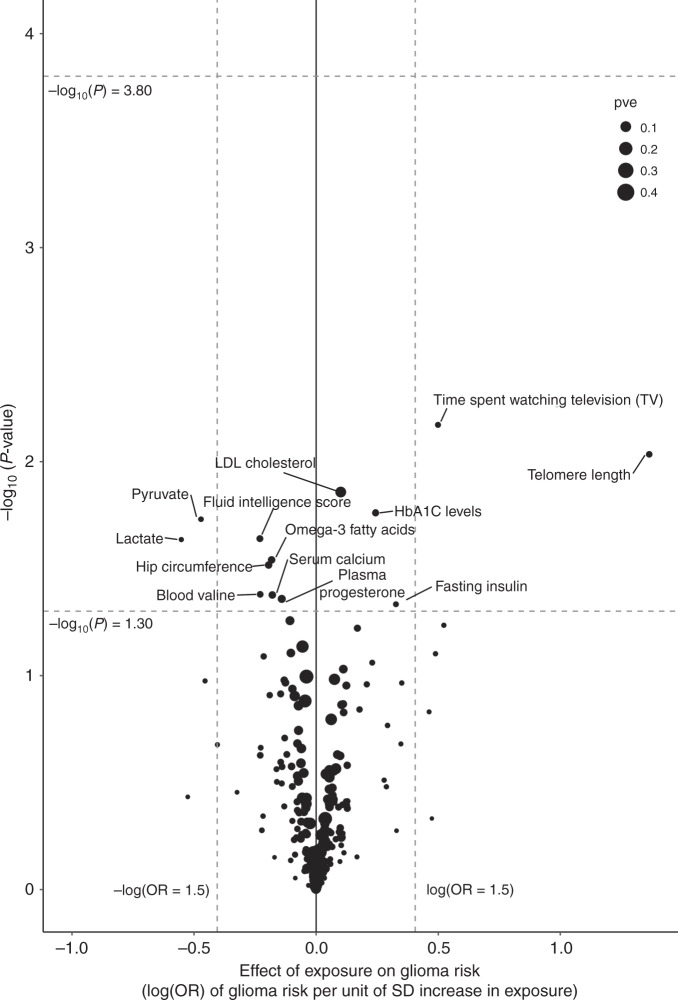
Volcano plot of the odds ratio per standard deviation from random-effects inverse-variance-weighted (IVW) Mendelian randomisation analysis of 316 phenotypes with risk of all glioma. Top dashed grey line corresponds to a Bonferroni-corrected *P* value of −log_10_ *P* value of 3.80 (1.58 × 10^−4^), indicating significant association. Bottom dashed grey line corresponds to −log_10_ *P* value of 1.30 (0.05), indicating a suggestive association. Vertical dashed grey lines correspond to log (OR = 1.5) and –log(OR = 1.5).

**Fig. 4:**
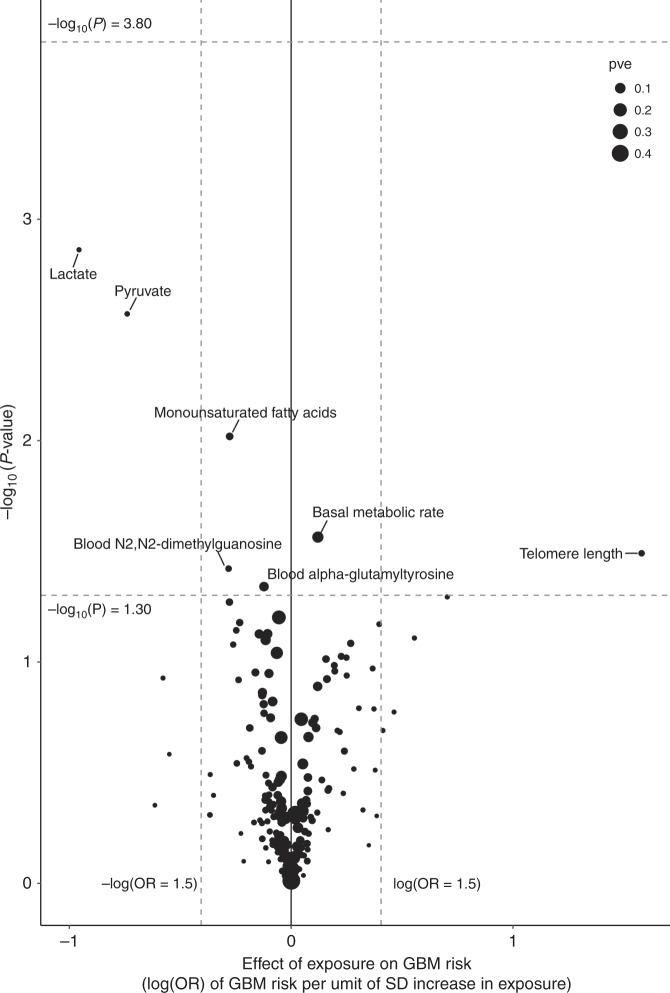
Volcano plot of the odds ratio per standard deviation from random-effects inverse-variance-weighted (IVW) Mendelian randomisation analysis of the 316 phenotypes with GBM risk. Top dashed grey line corresponds to a Bonferroni-corrected *P* value of −log_10_ *P* value of 3.80 (*P* = 1.58 × 10^−4^), indicating significant association. Bottom dashed grey line corresponds to −log_10_ *P* value of 1.30 (*P* = 0.05), indicating a suggestive association. Vertical dashed grey lines correspond to log (OR = 1.5) and –log (OR = 1.5).

**Fig. 5:**
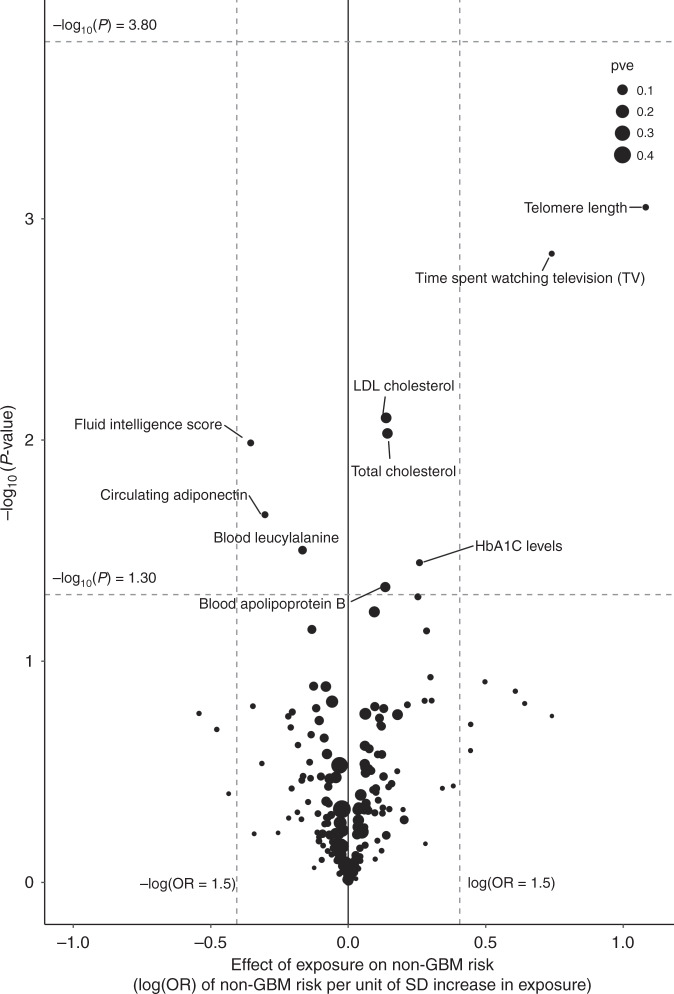
Volcano plot of the odds ratio per standard deviation from random-effects inverse-variance-weighted (IVW) Mendelian randomisation analysis of the 316 phenotypes with non-GBM risk. Top dashed grey line corresponds to a Bonferroni-corrected *P* value of −log_10_ *P* value of 3.80 (*P* = 1.58 × 10^−4^), indicating significant association. Bottom dashed grey line corresponds to −log_10_ *P* value of 1.30 (*P* = 0.05) indicating a suggestive association. Vertical dashed grey lines correspond to log (OR = 1.5) and –log (OR = 1.5).

The strength of the association between each of the 316 phenotypes studied and risk of all glioma, GBM and non-GBM tumours under IVW-RE models is shown in Figs. 3, 4 and 5, respectively, with the corresponding tabulated data in Supplementary Table A-C4. None of the traits showed a statistically significant association with risk of all glioma, GBM or non-GBM under an IVW-RE model. Thirteen traits showed suggestive evidence of association (*P* < 0.05) with risk of all glioma, but only leukocyte telomere length (LTL), serum low-density lipoprotein (LDL) cholesterol and plasma HbA1C levels showed consistent evidence of an association under the WME, WMBE and MR-RAP models (Supplementary Table A4).

**Fig. 6:**
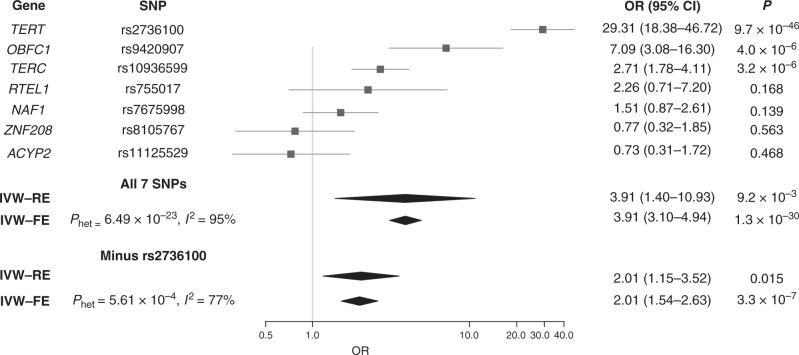
Forest plot showing the effect of alleles associated with longer leukocyte telomere length on all glioma risk. Diamonds represents overall causal effects estimated using both random and fixed-effects inverse-variance-weighted (IVW) models, both with and without the *TERT* SNP (rs2736100). Confidence intervals indicated by diamond width. Vertical line denotes the null value (OR_SD_ = 1).

Genetically increased LTL was associated with glioma risk; OR_s_ under IVW-RE and IVE-FE models’ respective ORs per SD were 3.91 (95% CI: 1.40–10.93, *P* = 9.24 × 10^–3^) and 3.91 (95% CI: 3.10–4.94, *P* = 1.29 × 10^−30^). The profound difference in strength of the relationship reflected the marked heterogeneity between the seven SNPs used as IVs (*P*_het_ = 6.49 × 10^−23^, *I*^2^ = 95%). The association was primarily driven by the *TERT* SNP (rs2736100), but also to a lesser extent of *TERC* (rs10936599) and *OBFC1* (rs9420907) SNPs (Fig. 6 and Supplementary Fig. 2). Excluding rs2736100 reduced heterogeneity (*P*_het_ = 5.61 × 10^−4^, *I*^2^ = 77%), but reduced the overall strength of the association (IVW-FE OR_SD_ = 2.01, 95% CI: 1.54–2.63, *P* = 3.29 × 10^−7^) (Supplementary Table A5).

Genetically predicted higher levels of LDL cholesterol were associated with an increased risk of glioma (IVW-RE: OR_SD_ = 1.11, 95% CI: 1.02–1.20, *P* = 1.39 × 10^−2^), but the association was largely reliant on rs7254892, with exclusion of rs7254892 association, under an IVW-RE model, reduced to OR_SD_ = 1.09 (95% CI: 1.00–1.18, *P* = 5.64 × 10^−2^). The association between genetically predicted higher HbA1C levels and glioma risk (IVW-RE: OR_SD_ = 1.28, 95% CI: 1.04–1.56, *P* = 1.73 × 10^−2^) was also largely dependent on a single SNP. The exclusion of rs16926246 reduced the association, under an IVW-RE model, to OR_SD_ 1.16 (95% CI: 0.93–1.46, *P* = 1.91 × 10^−1^).

Seven traits showed evidence of suggestive association (*P* < 0.05) with GBM risk under IVW-RE models (Supplementary Table B4). However, only the association between genetically determined increased LTL and GBM risk (IVW-RE: OR_SD_ = 4.86 (95% CI: 1.14–20.63, *P* = 3.23 × 10^−2^) and IVW-FE: OR_SD_ = 4.86 (95% CI: 3.65–6.46, *P* = 1.80 × 10^−27^)) was consistent under WME, WMBE and MR-RAP models (Supplementary Table B4). As with all glioma, there was marked heterogeneity between SNP associations (*P*_het_ = 9.19 × 10^−31^, *I*^2^ = 96%) with the association being driven by the *TERT* (rs2736100) SNP (Supplementary Table B5, Supplementary Figs. 3 and 5).

Nine traits showed evidence of a suggestive association (*P* < 0.05) with non-GBM risk under IVW-RE models; however, only genetically predicted serum LDL cholesterol and total cholesterol (TC) levels showed consistent evidence under WME, WMBE and MR-RAP models (Supplementary Table C4). Increased LDL cholesterol (IVW-RE: OR_SD_ = 1.15, 95% CI: 1.04–1.27, *P* = 7.94 × 10^−3^) and TC (IVW-RE: OR_SD_ = 1.15 (95% CI: 1.04–1.28, *P* = 9.33 × 10^−3^)) remained suggestively associated with increased non-GBM risk after leave-one-out analysis (Supplementary Table C5).

## Discussion

Despite its comparative rarity, glioma is one of the cancers of unmet need, given the significant morbidity and mortality associated with its diagnosis. Despite much research over the past three decades, we still know very little about the aetiological basis of glioma, which is a barrier to developing strategies to reduce disease burden. This contrasts markedly to the success of cohort and case–control studies of the common cancers, such as breast, lung and colorectal, which have identified major determinants of risk.^38,39^ Aside from the incidence of glioma limiting the power of any cohort study to demonstrate a causal association, case–control studies of glioma are more apt to suffer from bias than studies of other tumour types.^14^ It is entirely plausible that because of such biases, previous observational epidemiological studies have reported mixed results for the possible relationship between glioma and fat, cholesterol,^6^ BMI, physical activity,^7,40,41^ blood pressure^8^ and diabetes.^42^

MR can circumvent many limitations of a conventional observational study, and the methodology is therefore increasingly being used to examine the impact of interventions on disease risk. The value of MR has been greatly enhanced by the wealth of GWAS data now available on multiple traits, which provide SNPs that can be used as IVs. These data have allowed us to test the relationship between multiple traits and glioma risk in a hypothesis-free manner by performing a MR-PheWAS.

Genetically determined blood LTL has previously been associated with GBM risk, based on a 5% subset of the GWAS data we analysed.^19^ The association is principally driven by the *TERT, TERC* and *OBFC1* SNPs, whereas the *RTEL1, NAF1, ACYP2* and *ZNF208* SNPs show only limited support for an association (Supplementary Table A-C2, Supplementary Figs. 2–4). Since whole- blood TL is not strongly correlated with brain TL (pairwise correlation: 0.10–0.22), discordancy in IV SNPs could reflect tissue specificity.^43^ Our MR-PheWAS did not provide robust evidence for associations between glioma and any of the 316 phenotypes examined, which comprised traits relating to human behaviour, cognitive performance, physical body variations, metabolic factors and the immune system. We did however find support for raised LDL cholesterol, TC and HbA1C being associated with risk, relationships not observed in previous MR analyses of glioma.^16,18^ Our current analysis has, however, been able to leverage a greater number of SNPs as IVs, thereby increasing study power and enabling us to demonstrate the effects of smaller magnitudes that may have been missed by earlier work.

The strength of our MR study is the exploitation of large GWAS datasets to examine the relationship between multiple phenotypes and risk of glioma. Our analysis does, however, have limitations. Firstly, we were limited to studying phenotypes with genetic instruments available. Secondly, correcting for multiple testing inevitably means the potential for false negatives is not unsubstantial. Thirdly, even though we only considered traits for which the genetic instruments employed explained at least 0.1% of phenotypic variance, for a large number of traits, we still had limited power to demonstrate causal associations of a small effect.

While MR-PheWAS offers the ability to simultaneously evaluate a wide range of potential risk factors and avoid many of the biases and limitations of conventional epidemiological studies, it incurs the burden of multiple testing impacting on study power. Ongoing GWAS of glioma and subsequent meta-analyses are likely to greatly empower future MR-PheWAS. Furthermore, these large datasets offer the opportunity to pursue MR-PheWAS using ‘adaptive design' methodologies, which are increasingly used in clinical trials. A subset of outcome GWAS data would be analysed in stage 1, and only those exposures with a *P* value <0.05 would be evaluated using the remaining dataset, thereby potentially reducing the burden of dealing with multiple testing.^44,45^

In conclusion, our study provides further insight into the landscape of glioma aetiology and sheds light on factors for which the evidence from conventional epidemiological studies has been mixed. Specifically, we provide evidence against any of the 316 traits being major risk factors for the development of glioma, helping to deprioritise these factors in future studies. The advent of larger GWAS datasets of exposures and glioma offers the prospect of using MR-based strategies to search for possible causal associations with smaller effect sizes, potentially elucidating stronger associations with risk factors that currently only show a suggestive level of association.

## Supplementary information

Supplementary File list and figures

Combined Supplementary Tables: all glioma (A), GBM only (B) and nonGBM only (C) data.

## Data Availability

Genetic instruments can be obtained through MR-Base (http://www.mrbase.org/)^22^ or from the individual reference papers. Meta-analysed glioma GWAS data were obtained from the study by Melin et al.,^21^ which is a meta-analysis of eight independent GWAS studies (UK,^46^ French,^47^ German,^48^ MDA,^49^ UCSF-SFAGS,^49^ GliomaScan,^50^ GICC^51^ and UCSF/Mayo^52^) detailed in Supplementary Table A-C4. Genotype data from the Glioma International Case-Control Consortium Study GWAS are available from the database of Genotypes and Phenotypes (dbGaP) under accession phs001319.v1.p1. Genotypes from the GliomaScan GWAS can be accessed through dbGaP accession phs000652.v1.p1.
