# Supplementary material for: Searching for causal relationships of glioma: a phenome-wide Mendelian randomisation study
Source: Br J Cancer. 2020 Oct 6;124(2):447–54. doi: 10.1038/s41416-020-01083-1 (PMC7852872; doi:10.1038/s41416-020-01083-1)
Supplement: Supplementary file 1 — Supplementary File list and figures [file 41416_2020_1083_MOESM1_ESM.docx]

**SUPPLEMENTARY TABLES**

**Combined Supplementary Table: (A) All Glioma Data, (B) GBM Data and (C) Non GBM Data.** A, B and C datasets each have seven associated sub-tables with the following titles:

ST X1 - Modifiable risk factors included in MR analysis

ST X2 - Instrumental variable information: effect allele, effect allele frequency, effect on exposure, strength of association with outcome for SNP

ST X3 - Summary of eight Glioma GWAS used in meta-analysis

ST X4 - Causal estimates from Wald ratio, IVW-RE, MR-RAPS, Weighted Median Estimator, Weighted Mode Estimator and IVE-FE for each exposure and disease risk.

ST X5 - Leave-one-out analysis results for modifiable SNPs.

ST X6 - MR Egger results - assessing pleiotropy of exposures used.

ST X7 - Comparison of Cochran's Q and Rucker's Q' values

**SUPPLEMENTARY FIGURES**


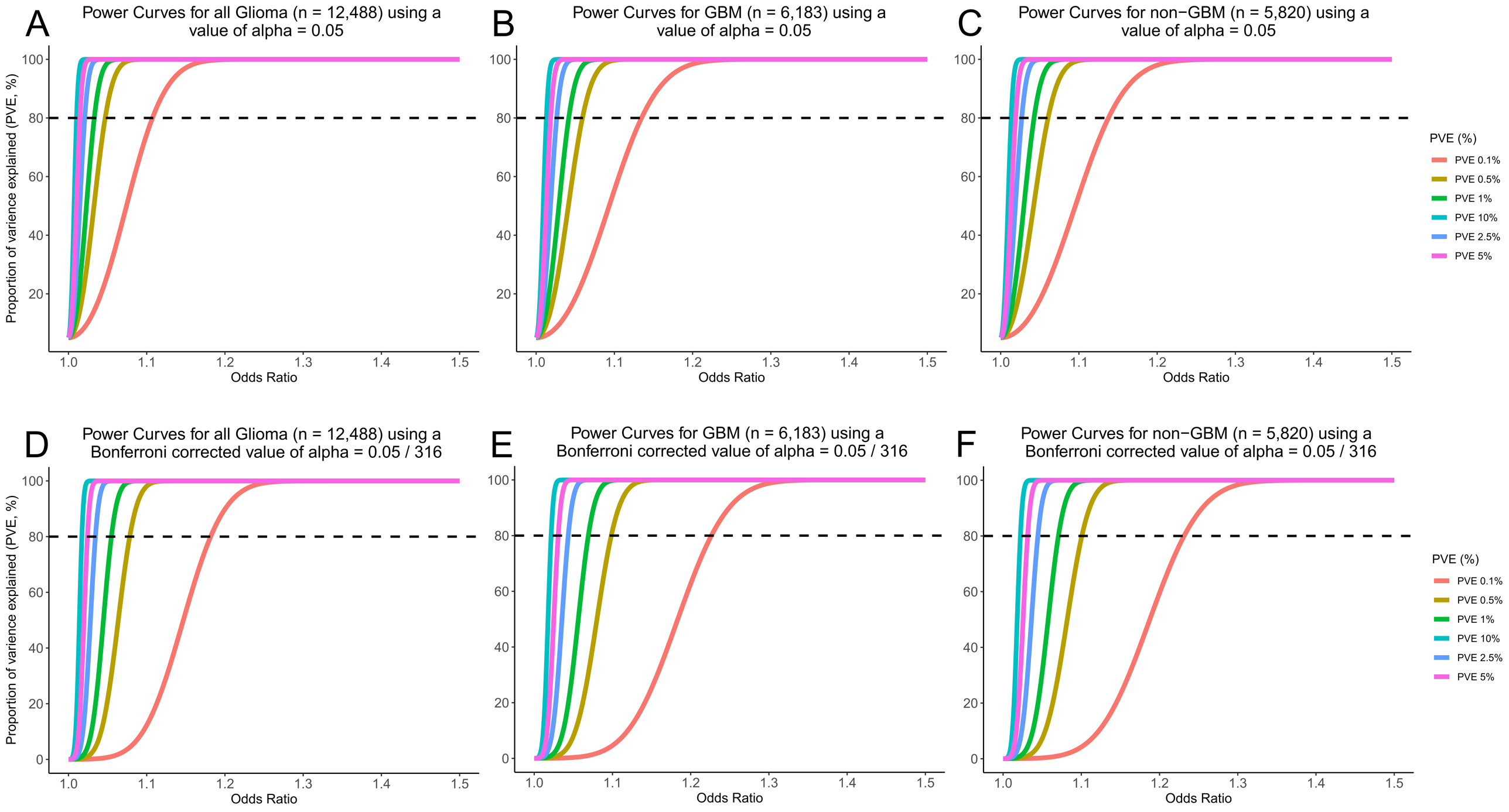


**Supplementary Figure 1: Power curves of the MR analysis to identify causal associations for (A) glioma, (B) GBM and (C) non-GBM at an alpha of 0.05.** **(D) glioma, (E) GBM and (F) non-GBM at an alpha of 0.05/316 (Bonferroni corrected value).**


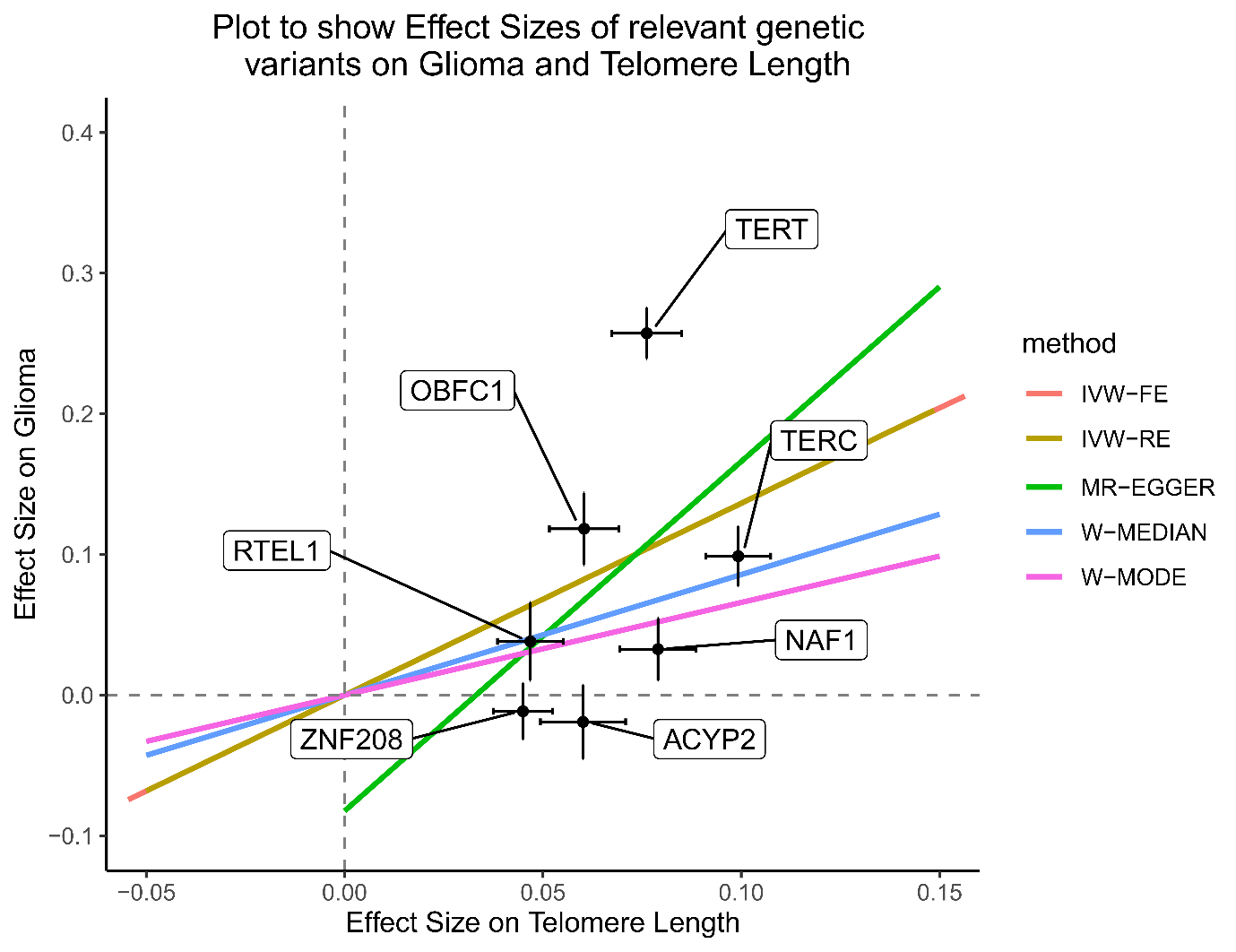


**Supplementary Figure 2: MR sensitivity analyses scatter plots.** MR sensitivity analyses, showing scatter plot of single nucleotide polymorphism genetic variant regression coefficients (effect sizes) quantifying the level of association of the exposure (leukocyte telomere length) with the glioma risk in an MR analysis. Solid lines represent regression coefficients using different models.

**
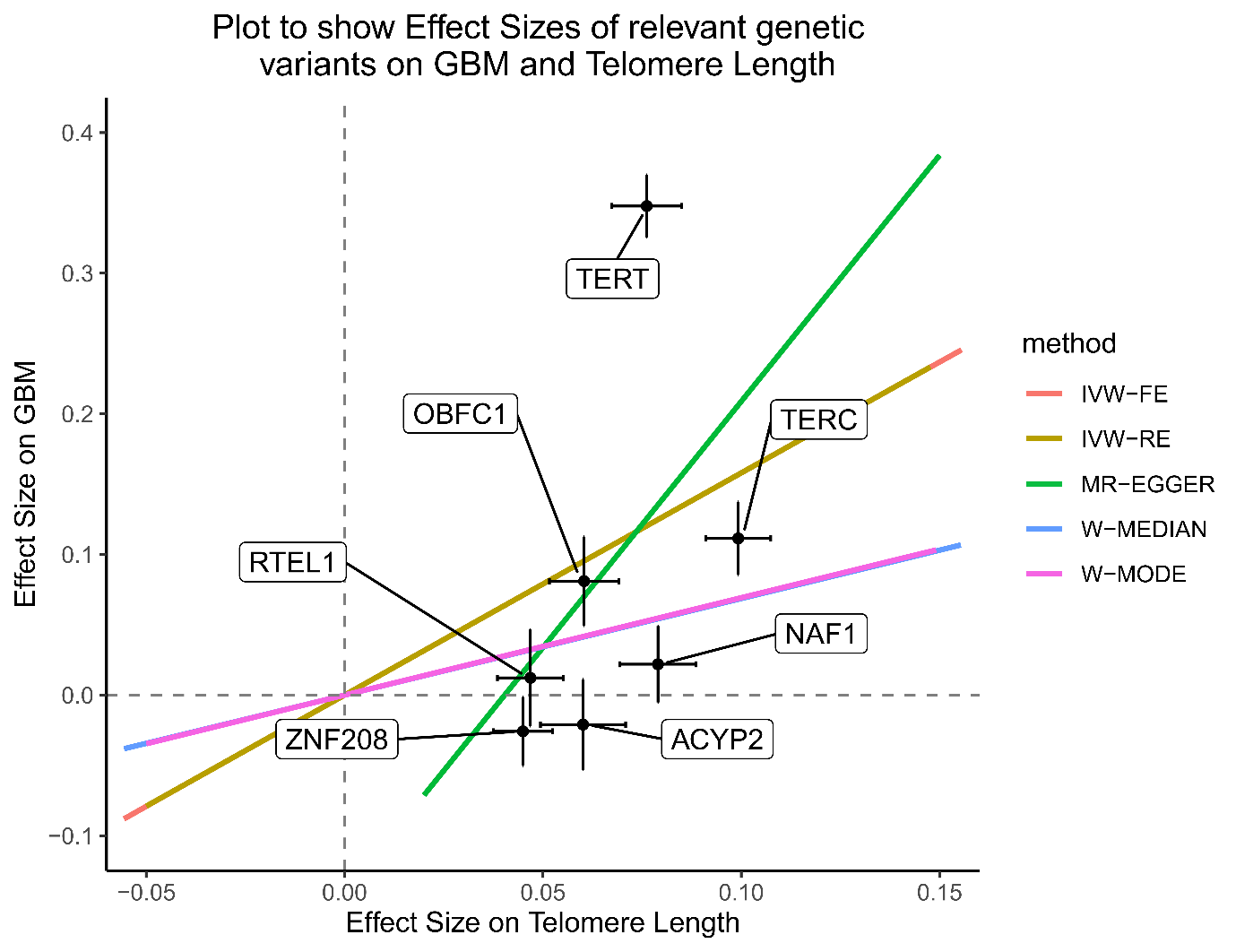
**

**Supplementary Figure 3: MR sensitivity analyses scatter plots.** MR sensitivity analyses, showing scatter plot of single nucleotide polymorphism genetic variant regression coefficients (effect sizes) quantifying the level of association of the exposure (leukocyte telomere length) with GBM risk in an MR analysis. Solid lines represent regression coefficients using different models.

**
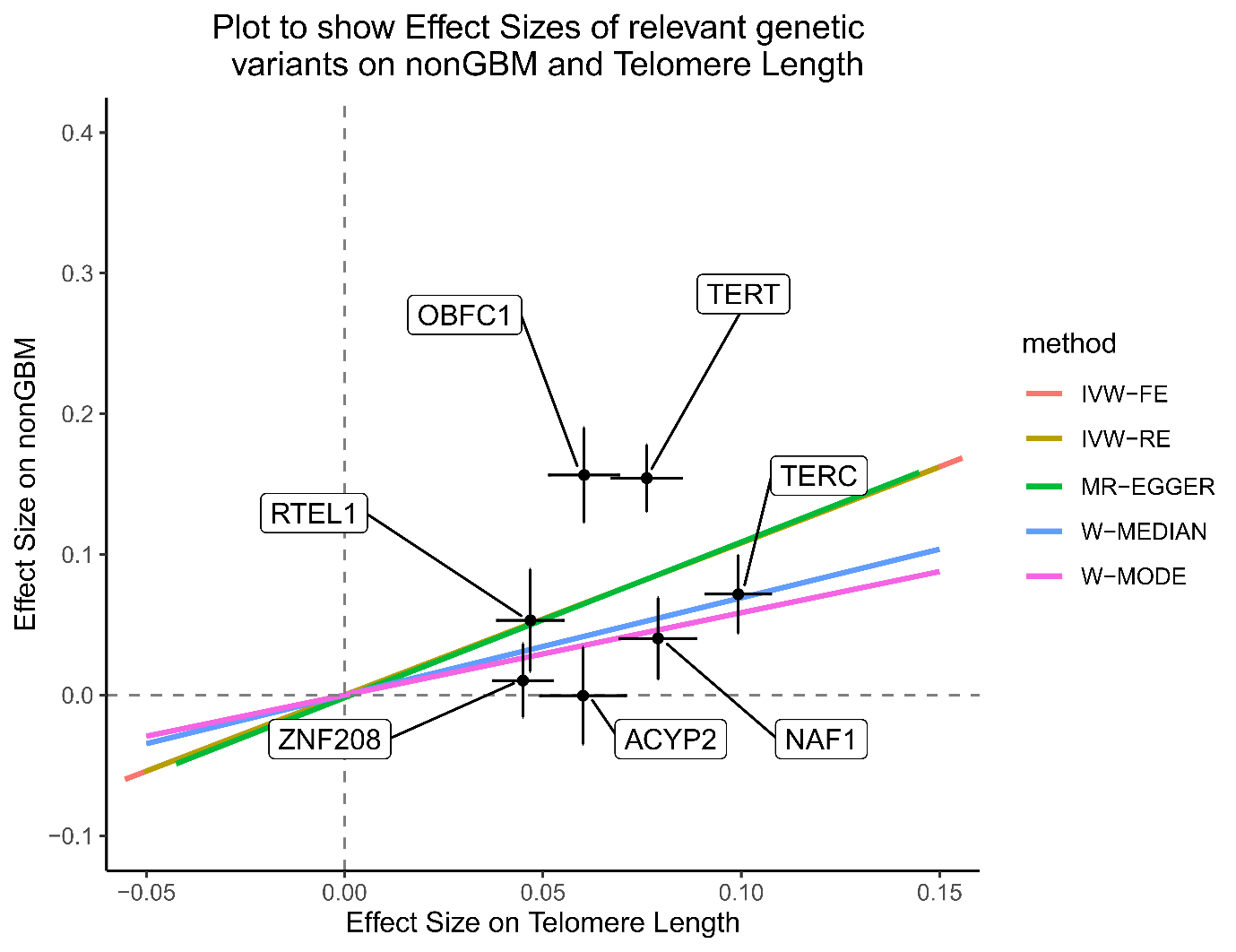
**

**Supplementary Figure 4: MR sensitivity analyses scatter plots.** MR sensitivity analyses, showing scatter plot of single nucleotide polymorphism genetic variant regression coefficients (effect sizes) quantifying the level of association of the exposure (leukocyte telomere length) with non-GBM respectively risk in an MR analysis. Solid lines represent regression coefficients using different models.


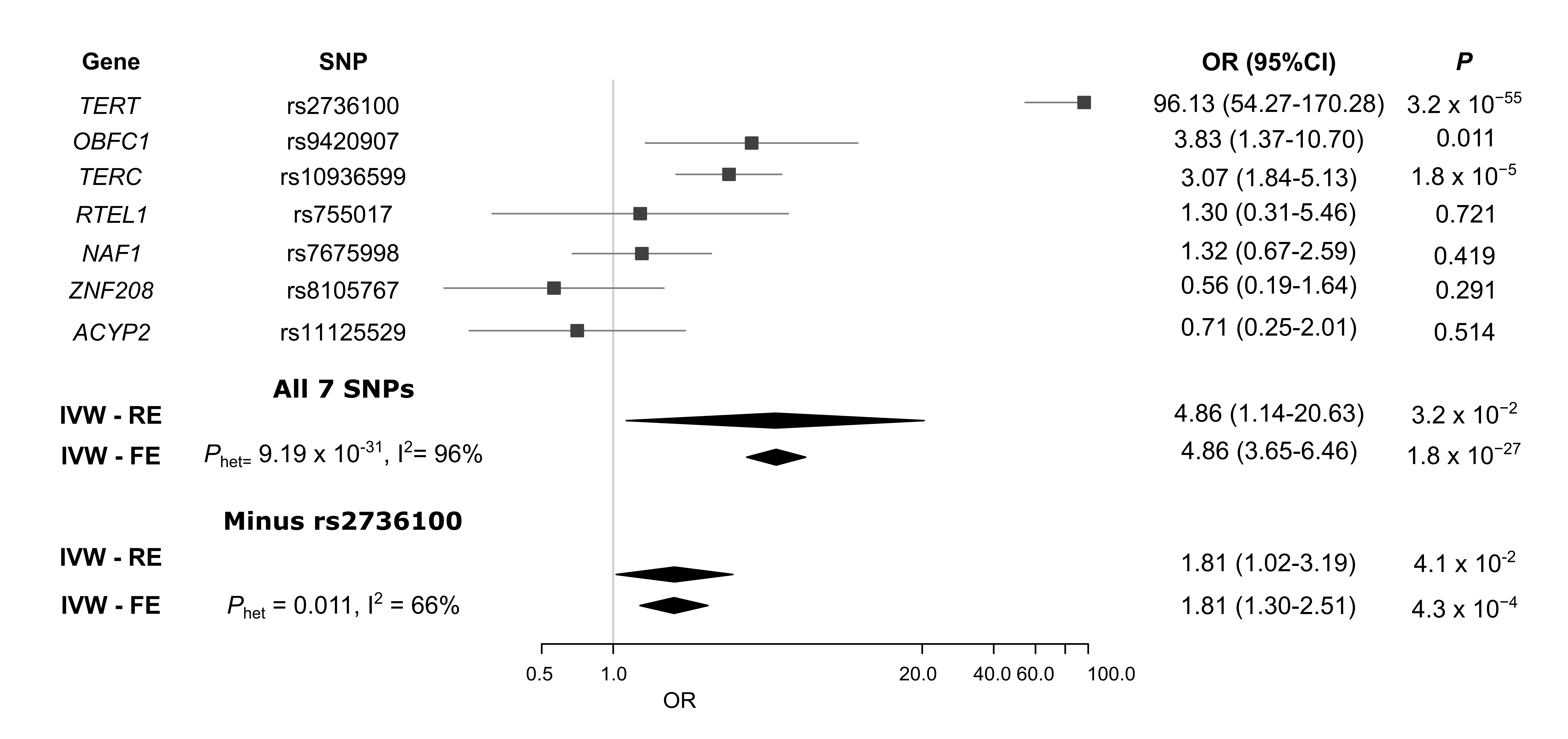


**Supplementary Figure 5: Forest plot showing the effect of alleles associated with longer leukocyte telomere length on all GBM risk.** Diamonds represents overall causal effects estimated using both random and fixed-effects inverse variance weighted models, both with and without the *TERT* SNP (rs2736100). Confidence intervals indicated by diamond width. Vertical line denotes the null value (OR_SD_ = 1).

**
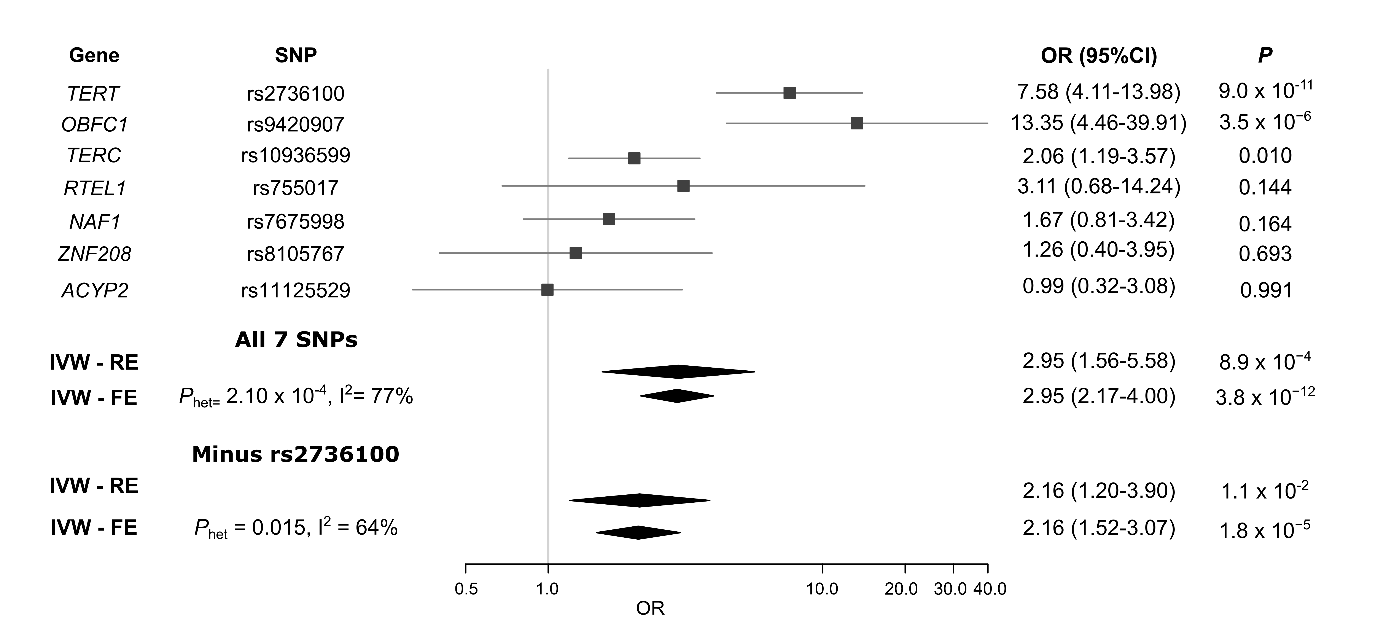
Supplementary Figure 6: Forest plot showing the effect of alleles associated with longer leukocyte telomere length on all non-GBM risk.** Diamonds represents overall causal effects estimated using both random and fixed-effects inverse variance weighted models, both with and without the *TERT* SNP (rs2736100). Confidence intervals indicated by diamond width. Vertical line denotes the null value (OR_SD_ = 1).

**
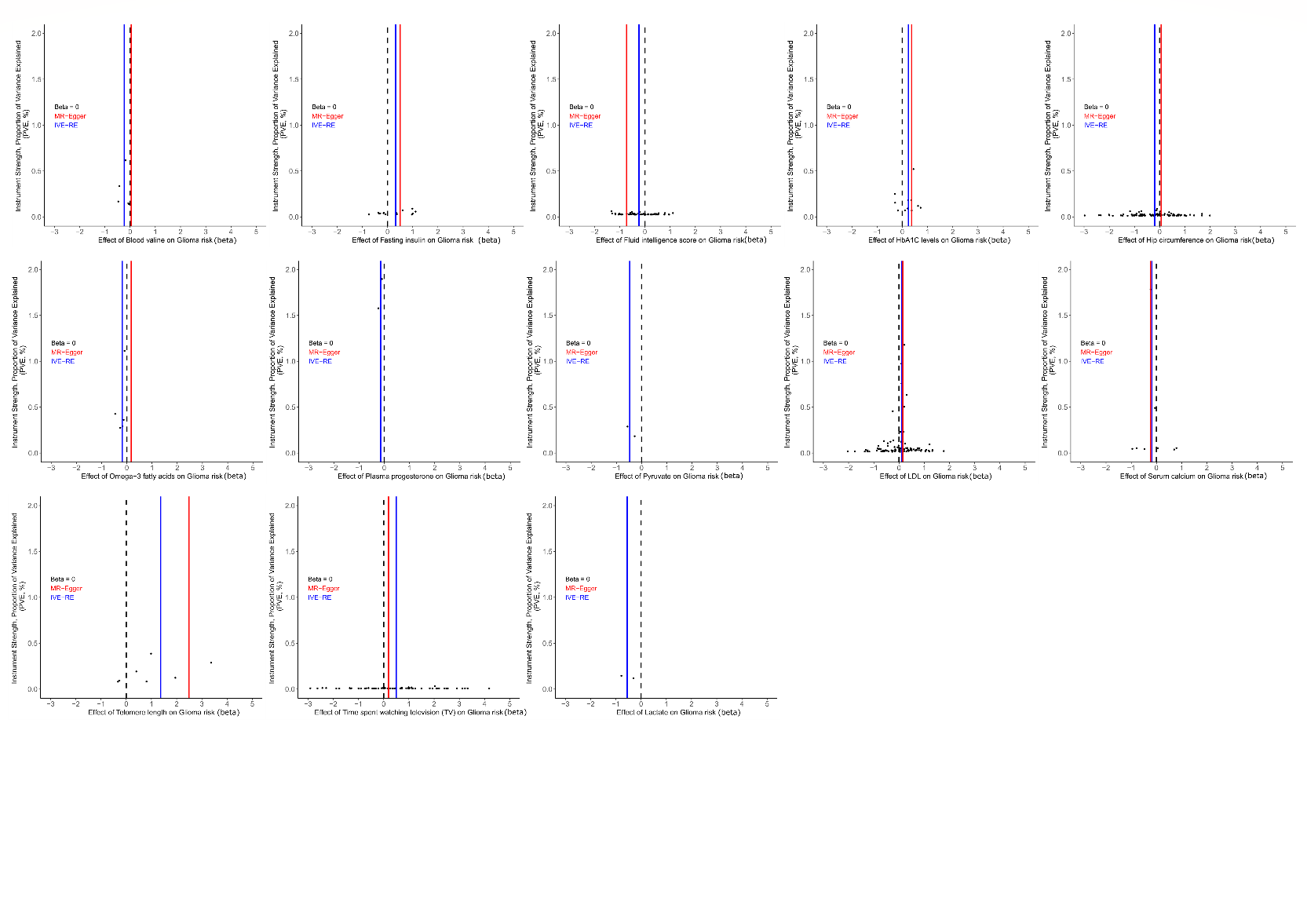
**

**Supplementary Figures 7: Funnel plots for all exposures showing at least a suggestive association with glioma.** Black dots represent each genetic variants’ effect (beta), used for each respective trait on glioma risk. Y-axis shows each genetic variants strength, shown as proportion of variance explained (PVE, %). Dashed black line shows beta = 0. Red and blue lines show MR-Egger and IVW-RE beta values respectively.


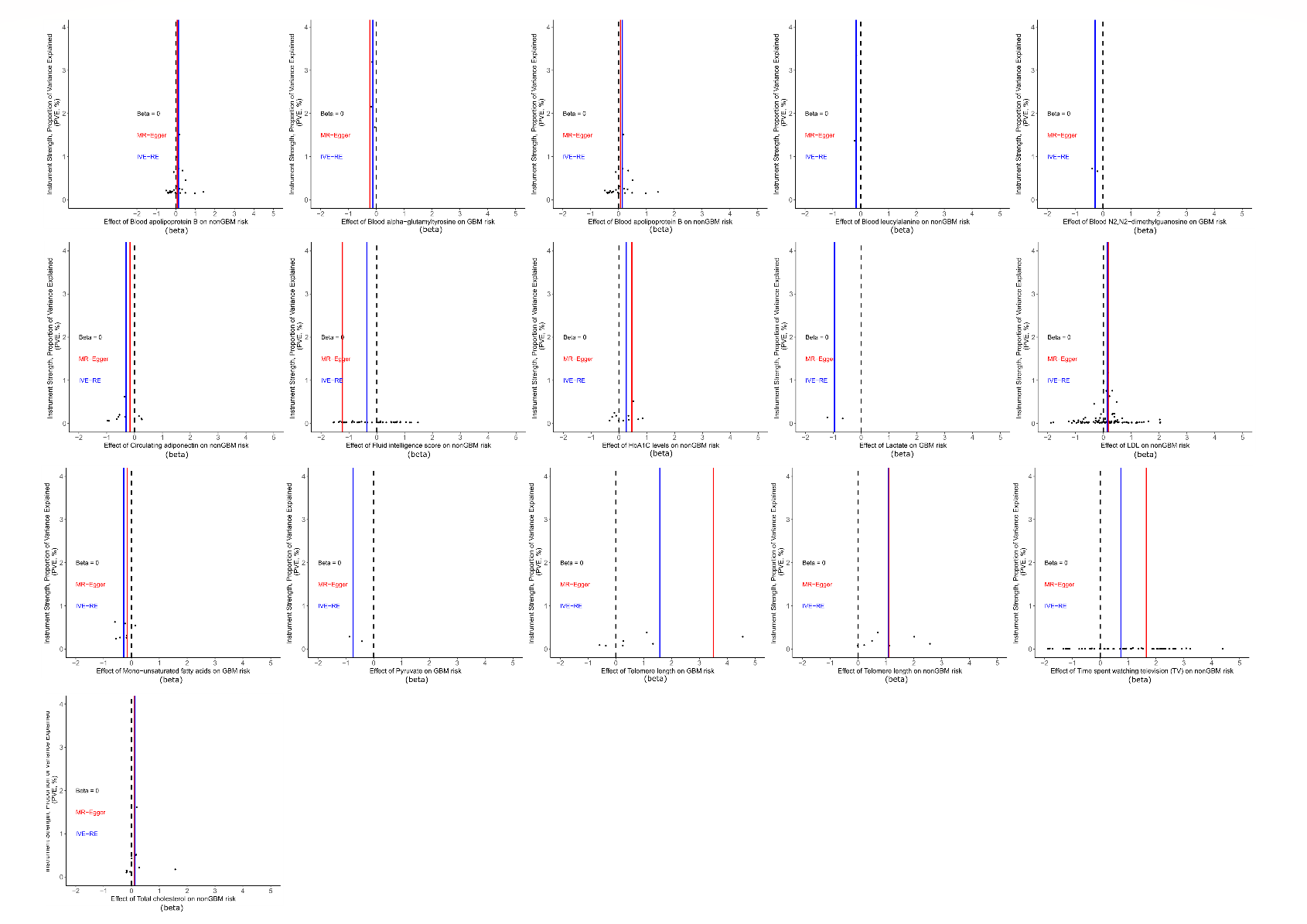


**Supplementary Figures 8: Funnel plots for all exposures showing at least a suggestive association with GBM.** Black dots represent each genetic variants’ effect (beta), used for each respective trait on GBM risk. Y-axis shows each genetic variants strength, shown as proportion of variance explained (PVE, %). Dashed black line shows beta = 0. Red and blue lines show MR-Egger and IVW-RE beta values respectively.


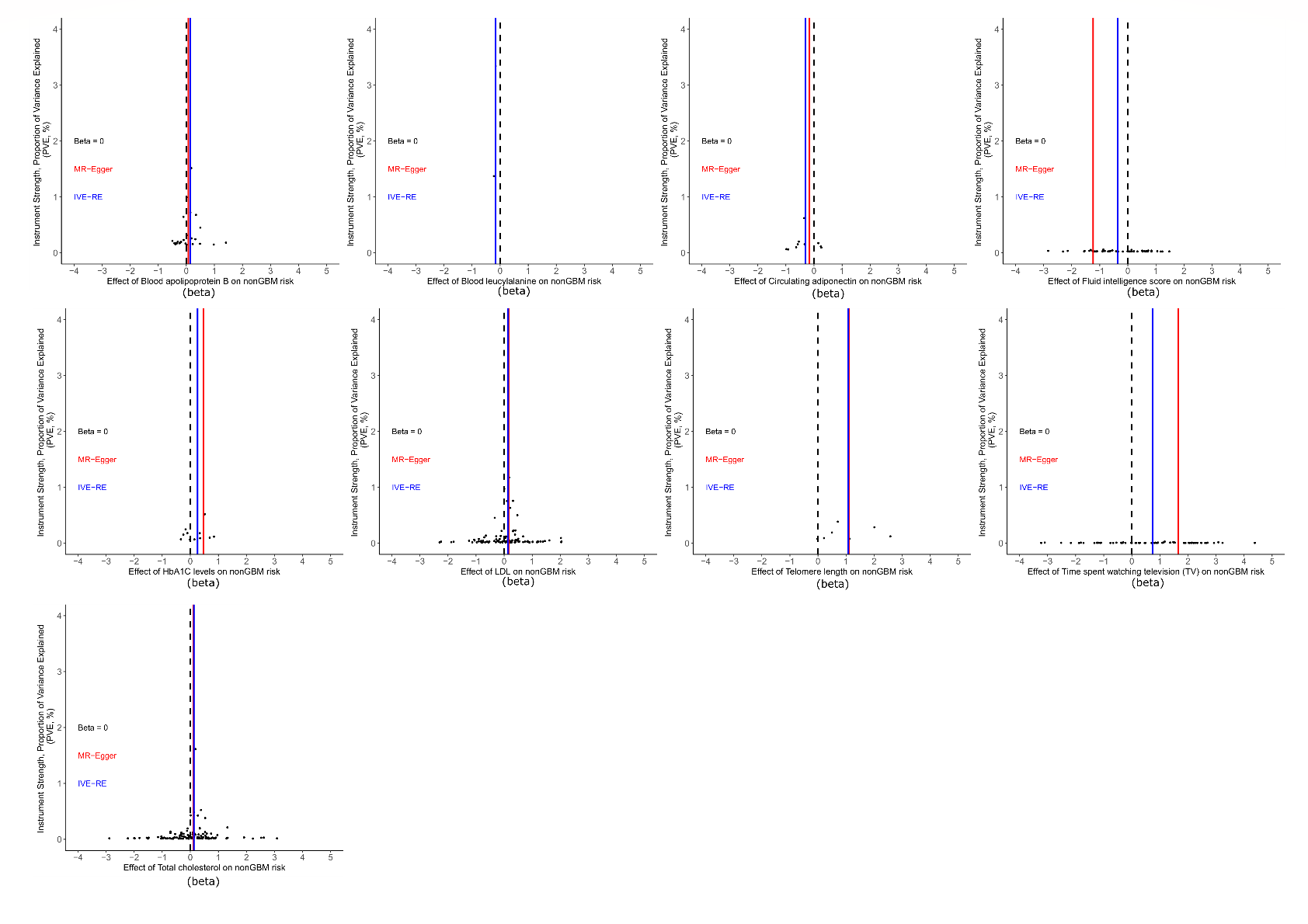


**Supplementary Figures 9: Funnel plots for all exposures showing at least a suggestive association with non-GBM.** Black dots represent each genetic variants’ effect (beta), used for each respective trait on non-GBM risk. Y-axis shows each genetic variants strength, shown as proportion of variance explained (PVE, %). Dashed black line shows beta = 0. Red and blue lines show MR-Egger and IVW-RE beta values respectively.
